# Safe and legal abortion access for survivors of rape and incest in West and Central Africa: a comparative case study analysis of key barriers

**DOI:** 10.1080/26410397.2026.2735510

**Published:** 2026-09-23

**Authors:** Mena Komi Agbodjavou, Harriët Meiborg, Anissa Eyi-Tayo Abdoulaye, Dorcas A. Delali Essilivi, Akioua William Nignan, Ulrich Valdo Yonga, Joseph Similoluwa Olujobi, Betinou Marie-Andrée Kadimon, Jeannette Bibiche Afounde, Rachidatou Compaoré, Abram Amétépé Agossou, Michel Edoukou N’Da-Ezoa, Moustapha Moncher Nsangou, Kéfilath Bello, Christelle Boyi Hounsou, Jonna Both, Jean-Paul Dossou

**Affiliations:** a Chief of Staff and Operations Facilitator, Centre de recherche en reproduction humaine et en démographie, Cotonou, Benin. *Correspondence*: magbodjavou@yahoo.com; b Research Assistant, Rutgers, Utrecht, The Netherlands; c Training Implementation Coordination Assistant, Centre de recherche en reproduction humaine et en démographie, Cotonou, Benin; d Junior Researcher, Centre de recherche en reproduction humaine et en démographie, Lomé, Togo; e Junior Researcher, Centre de recherche en reproduction humaine et en démographie, Ouagadougou, Burkina Faso; f Junior Researcher, Centre de recherche en reproduction humaine et en démographie, Yaoundé, Cameroon; g Junior Researcher, Centre de recherche en reproduction humaine et en démographie, Cotonou, Benin; h Junior Researcher, Centre de recherche en reproduction humaine et en démographie, Abidjan, Côte d’Ivoire; i Independent Consultant, Centre de recherche en reproduction humaine et en démographie, Yaounde, Cameroon; j Independent Consultant, Centre de recherche en reproduction humaine et en démographie, Ouagadougou, Burkina Faso; k Independent Consultant, Centre de recherche en reproduction humaine et en démographie, Lome, Togo; l Independent Consultant, Centre de recherche en reproduction humaine et en démographie, Abidjan, Côte d’Ivoire; m Independent Consultant, Centre de recherche en reproduction humaine et en démographie, Yaounde, Cameroon; n Head of the Health Policy and Systems Department, Centre de recherche en reproduction humaine et en démographie, Cotonou, Benin; o Head of the Department of Biostatistics, Epidemiology, Disease Prevention and Control, Centre de recherche en reproduction humaine et en démographie, Cotonou, Benin; p Researcher, Rutgers, Utrecht, The Netherlands; q Executive Director, Centre de recherche en reproduction humaine et en démographie, Cotonou, Benin

**Keywords:** safe and legal abortion access, sexual violence, survivors, implementation barriers, Maputo Protocol, West and Central Africa, comparative case study

## Abstract

Despite legal provisions allowing for abortion in cases of rape and incest, survivors in West and Central Africa still face many access barriers. Understanding these obstacles is vital for their rights and health. This study looked at barriers to safe and legal abortion services for survivors of sexual violence in five West and Central African countries. We did a comparative case study in Benin, Burkina Faso, Cameroon, Côte d’Ivoire, and Togo. Data collection included documentary analysis, 164 semi-structured interviews with institutional actors, civil society members, community leaders, and sexual violence survivors, along with 14 focus group discussions and a multi-level analytical framework. We applied a four-phase collaborative qualitative thematic analysis. This helped us identify barriers at macro (policy), meso (organisational), and micro (individual) levels. The analysis reveals how interconnected barriers restrict access. At the macro level, obstacles include unclear laws, missing regulations, and bans on abortion-related communication. Meso-level barriers feature inconsistent institutional practices, unprepared health facilities, and poor coordination. Micro-level challenges involve limited awareness of legal rights, widespread social and religious stigma, and informal conscientious objection practices. For survivors seeking to end the pregnancy, these barriers lead to unsafe abortion or forced continuation of pregnancy. Legal provisions alone do not guarantee survivors’ access to safe and legal abortion. Coordinated efforts are needed to address legal, institutional, and social obstacles, transforming legal rights into real access and reducing unsafe abortion practices.

## Plain language summary

In five West and Central Africa countries (Benin, Burkina Faso, Cameroon, Côte d’Ivoire, and Togo), the law lets women who become pregnant from rape or incest end the pregnancy safely. Yet most survivors cannot get this care. This study asked why. The research team interviewed 164 people (doctors, nurses, judges, police officers, policy makers, community leaders, and survivors of sexual violence) and held 14 focus group discussions. They also reviewed each country’s laws and policies. The study found barriers at three levels. In laws and policy, rules and guidelines are often unclear, and procedures take too long. Health workers and survivors alike do not always know survivors’ rights. In services, many health workers refuse to help because of personal beliefs, and health, police, and court services do not coordinate well. In communities, stigma and silence surround both sexual violence and abortion. Survivors are often blamed rather than supported and families pressure them to keep the pregnancy. Many people do not know that safe abortion is legal in these cases. When survivors cannot get safe abortion care, they may turn to dangerous methods or be forced to continue an unwanted pregnancy. These are both harmful to their health and rights. Laws alone are not enough. Clearer laws, stronger leadership and implementation strategies, trained and supported health workers, communities informed about survivors’ rights, and support for survivors seeking care and justice must all come together to give survivors the care they need.

## Résumé

Malgré les dispositions légales autorisant l’avortement en cas de viol et d’inceste, les victimes en Afrique centrale et de l’Ouest rencontrent encore de nombreux obstacles pour accéder aux services. Il est essentiel de comprendre ces obstacles pour les droits et la santé de ces femmes. L’étude a examiné les obstacles que rencontrent les victimes de violence sexuelle pour avoir accès à des services d’avortement sûrs et légaux dans cinq pays d’Afrique centrale et de l’Ouest. Nous avons mené une étude de cas comparative au Bénin, au Burkina Faso, au Cameroun, en Côte d’Ivoire et au Togo. Le recueil des données comprenait une analyse documentaire, 164 entretiens semi-structurés avec des acteurs institutionnels, des membres de la société civile, des dirigeants communautaires et des personnes ayant subi des violences sexuelles, ainsi que 14 discussions de groupe. Nous avons appliqué une analyse thématique qualitative collaborative en quatre phases ainsi qu'un cadre d'analyse multiniveaux. Cela nous a permis d’identifier les obstacles aux niveaux macro (politiques), méso (organisationnel) et micro (individuel). L’analyse a révélé comment des obstacles interdépendants restreignent l’accès. Au niveau macro, les obstacles comprennent des lois imprécises, une règlementation inexistante et des interdictions de communiquer sur l’avortement. Les obstacles au niveau méso se rapportent à l’incohérence des pratiques institutionnelles, à la préparation insuffisante des centres de santé et à une mauvaise coordination. Au nombre des difficultés rencontrées au niveau micro figurent une conscience limitée des droits reconnus, une stigmatisation sociale et religieuse généralisée et des pratiques d’objection de conscience informelles. Pour les victimes souhaitant interrompre leur grossesse, ces obstacles conduisent à un avortement non médicalisé ou à la poursuite forcée de la grossesse. Les dispositions juridiques ne garantissent pas à elles seules l’accès à un avortement sûr et légal. Des efforts concertés sont nécessaires pour lever les obstacles juridiques, institutionnels et sociaux, transformer les droits en accès véritable et réduire les pratiques d’avortement non médicalisé.

## Resumen

A pesar de las disposiciones legislativas que permiten el aborto en casos de violación e incesto, las sobrevivientes en África Occidental y Central aún enfrentan muchas barreras al acceso. Entender estos obstáculos es vital para sus derechos y su salud. Este estudio examinó las barreras a los servicios de aborto seguro y legal para sobrevivientes de violencia sexual en cinco países de África Occidental y Central. Realizamos un estudio comparativo de casos en Benín, Burkina Faso, Camerún, Costa de Marfil y Togo. La recolección de datos consistió en análisis documental, 164 entrevistas semiestructuradas con actores institucionales, miembros de la sociedad civil, líderes comunitarios y sobrevivientes de violencia sexual, así como 14 grupos focales. Aplicamos un análisis temático cualitativo y colaborativo de cuatro fases y un marco analítico multinivel. Esto nos ayudó a identificar las barreras a nivel macro (políticas), meso (organizacional) y micro (individual). El análisis revela la manera en que las barreras interconectadas restringen el acceso. A nivel macro, los obstáculos eran leyes ambiguas, ausencia de normas y prohibición de la comunicación relacionada con el aborto. A nivel meso, las barreras eran prácticas institucionales incongruentes, establecimientos de salud no preparados y mala coordinación. A nivel micro, los retos consistían en conocimiento limitado de los derechos jurídicos, estigma social y religioso generalizados y prácticas de objeción de conciencia informales. Debido a estas barreras, las sobrevivientes que buscaban interrumpir su embarazo se vieron obligadas a recurrir a un aborto inseguro o a continuar su embarazo. Las disposiciones legislativas por sí solas no garantizan el acceso de las sobrevivientes al aborto seguro y legal. Se necesitan esfuerzos coordinados para abordar los obstáculos legislativos, institucionales y sociales, transformar los derechos jurídicos en acceso real y reducir las prácticas de aborto inseguro.

## Introduction

Few countries in sub-Saharan Africa permit abortion on request for women and girls. The protocol of the African Charter on Human and Peoples’ Rights on the Rights of Women in Africa (the Maputo Protocol), in its article 14(2)(c), calls on States to protect women’s reproductive rights by authorising abortion in cases of sexual assault, rape, incest, and where the continued pregnancy endangers the mental or physical health or life of the woman or the fetus.^1^ Most countries in sub-Saharan Africa maintain heavily restrictive abortion laws, permitting the procedure only in cases of medical emergencies or pregnancies resulting from rape and incest, in partial alignment with the Protocol. Despite these constraints, given sub-Saharan Africa’s high rates of sexual and gender-based violence (SGBV), including the steep increases observed in conflict settings, these legal provisions potentially apply to substantial numbers of women and girls. Therefore, it is crucial to examine whether the existing legal frameworks that permit abortion in cases of rape and incest effectively protect the rights of survivors in practice.

Sexual and gender-based violence affects one in three women in sub-Saharan Africa.^2^ Precise country-specific estimates of pregnancies resulting from sexual violence remain limited because of underreporting. A multi-country analysis of Demographic and Health Survey data from 22 sub-Saharan African countries found that sexual violence was significantly associated with mistimed and unwanted pregnancies.^3^

Research consistently documents a persistent gap between legal provisions and effective access to safe and legal abortion services in sub-Saharan Africa. Legal authorisation does not guarantee women's access to these services due to stigmatisation and complex legal requirements.^4,5^ For example, in cases of pregnancies resulting from rape, several countries require extensive documentation, including magistrate attestations (in Zimbabwe) and a public prosecutor certificate (in Cameroon and Burkina Faso).^6^ Studies further reveal that sexual violence survivors face significant barriers to reporting rape or incest, especially when perpetrators are family members or relatives, effectively precluding access to formal care systems.^7^ Survivors may also face stigma linked to the sexual violence itself, including shame, blame, and fear of social rejection, which can further delay disclosure and help-seeking.^8^ Furthermore, there is significant social stigma associated with abortion since it clashes with cultural and religious norms that emphasise the importance of fertility and the belief that life starts at conception.^7^ These overlapping stigmas may further complicate timely access to formal care.

Research confirms that effective access to safe abortion varies significantly across socio-demographic characteristics, with certain groups experiencing intersectional vulnerability that compounds their marginalisation.^9,10^ Adolescents (15–19 years) may be especially vulnerable to barriers in access to safe and legal abortion because of limited contraceptive use, restricted access to sexuality education and reproductive health services, and gender norms that may constrain them more strongly because of age-related dependency, limited autonomy, and intensified social control.^6^

Despite the persistently high burden of unsafe abortion and sexual violence in sub-Saharan Africa, these phenomena are typically examined separately, with limited research addressing their interconnections. Although emerging evidence links sexual violence to mistimed and unwanted pregnancy, few studies examine how this chain unfolds in practice through abortion trajectories, and even fewer analyse how legal exceptions for rape and incest translate, or fail to translate, into effective access to safe and legal abortion in specific country contexts.^3,4,6,11^ Qualitative evidence from Cameroon, one of the countries included in this study, further documents pregnancies following rape or sexual coercion that ended in unsafe abortion through informal care pathways, in a context where legal eligibility did not translate into effective access to care.^11^ Additionally, studies employing multi-level analytical approaches that examine various implementation levels remain scarce. Comparative studies are therefore needed to examine how different legal and policy environments shape survivors’ access in practice, and to distinguish shared barriers from those produced by specific legal and institutional configurations.

This article examines access to safe and legal abortion for survivors of sexual violence (rape and/or incest) in five countries with varying legal trajectories: Benin, Burkina Faso, Cameroon, Côte d’Ivoire, and Togo. It investigates whether survivors can access safe and legal abortion services when they decide it is their best option given their circumstances. Concretely, the study examines the existing legal frameworks, identifies implementation barriers across macro, meso, and micro levels, and analyses how these barriers are experienced along survivors’ care pathways. Understanding the barriers in the implementation of existing laws through comprehensive research is essential for informing policymakers and stakeholders while developing effective strategies that ensure survivors receive timely appropriate care.^12,13^

## Methodology

### Study design

This research compares access to safe and legal abortion for rape and incest survivors in five countries. The specific situation in each country is first analysed as an in-depth case study and then compared with the others to identify factors that explain observed similarities and differences. The units of analysis are Benin, Burkina Faso, Cameroon, Côte d’Ivoire, and Togo. Since complex social phenomena such as access to reproductive rights require careful contextual examination, case studies are particularly appropriate, especially when boundaries between issues and context are not clear.^14^

### Conceptual framework

This research employs two complementary frameworks to analyse the disconnect between policy intentions and effective safe and legal abortion service access for survivors. The first is Berman’s implementation framework (Figure 1), developed by Paul Berman^15^ and adapted by Dossou et al.^16^ The framework guided the comparative analysis across macro, meso, and micro levels. It distinguishes three stages, through which policies formulated by national or central authorities reach rights holders on the ground. At each stage, policies may encounter various scenarios supporting or undermining (through negative distortion or blockage) the initial political intention. These stages consist of the following:

- The macro-level of administration. This stage encompasses the decision-making phase through the establishment of concrete programmes for policy implementation. This involves macro-level stakeholders, including the Council of Ministers, resulting in decisions regarding the articulation between policy and resources aimed at translating intentions into concrete outcomes for rights-holders. These resources include human actors – natural or legal persons (through specification assignments and institutional arrangements) – financial resources, information resources (data, guides, protocols, procedures manuals, etc.) and implementation deadlines.
- The meso level of organisational adoption constitutes the second implementation stage, encompassing the transition of programme to local adoption at the operational service level. Discrepancies between programme directives and actual implementation may emerge at this level. This phase produces the organisational approach to implementing programme or policy provisions. Organisations can adopt policies in four ways: (i) non-adoption, (ii) co-option (adapting programmes to match existing practices), (iii) technical learning (adapting routine practices to incorporate policy requirements), or (iv) mutual adaptation, where both policy and service delivery are modified to ensure optimal implementation.
- The micro-level concerns programme or policy implementation by individual agents within organisations who interact directly with rights holders to deliver services. These agents function as street-level bureaucrats. While all stages are important, the transition to micro-implementation is critical because it directly determines the lived experience of the rights holders.

**Figure 1.**
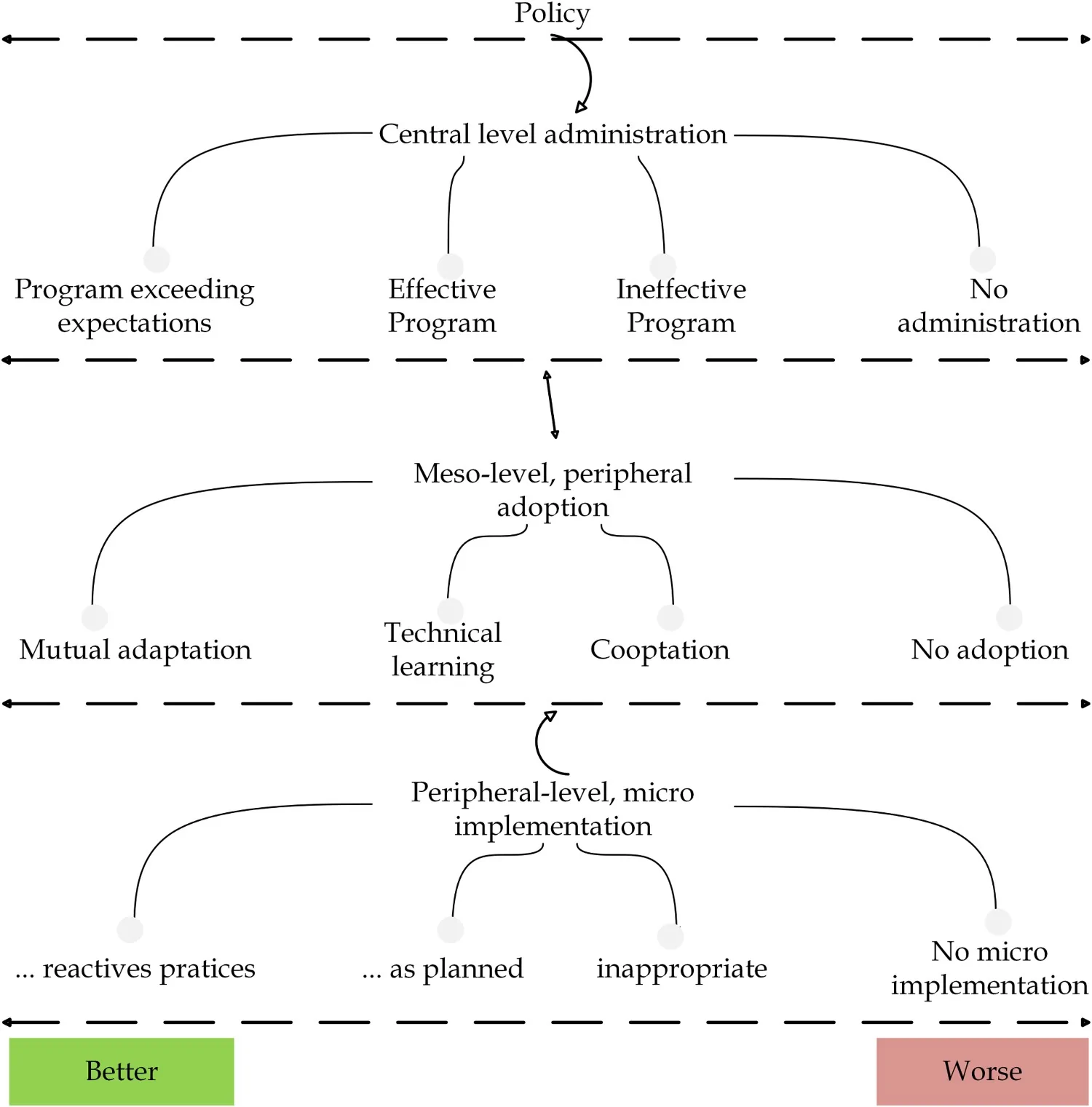
Adapted Berman’s implementation framework Note: This framework illustrates the multi-level implementation process, from policy formulation through central administration, meso-level adoption, to peripheral-level micro-implementation. The “Better to Worse” axis indicates relative performance across implementation modalities (e.g. “Program exceeding expectations”, “Effective Program”, “Ineffective Program”, “No administration”) Source: Adapted by Dossou et al. (2021) from Berman’s implementation framework.

The second framework employs the survivor’s pathway developed by the *Centre de recherche en reproduction humaine et en démographie* (CERRHUD) (Figure 2). This framework reconstructs the step-by-step journey that sexual violence survivors are expected to follow according to the laws and procedures to access safe and legal abortion, from information and disclosure to reporting, medical certification, judicial authorisation, care, and follow-up. In this study, it was used as a complementary interpretive framework to inform the development of interview and focus group guides, help identify the categories of actors involved at different stages of the pathway, and analyse pathway ruptures and delays experienced by survivors, especially at the micro level.

**Figure 2.**
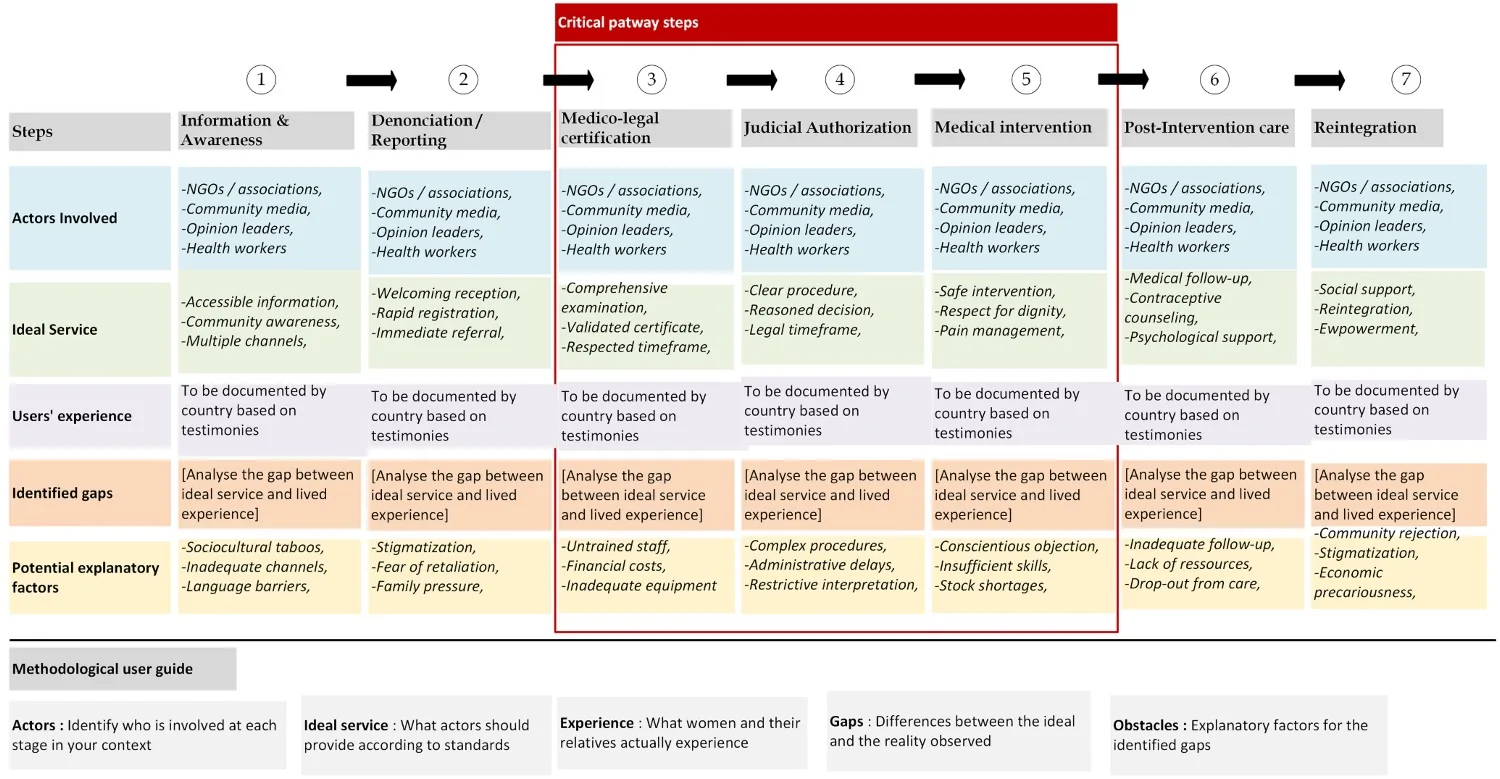
Service line model across the abortion care pathway Note: This diagram presents the ideal service delivery model across seven stages of the abortion care pathway: Information & Awareness, Reporting, Medico-legal Certification, Judicial authorisation, Medical Intervention, Post-Intervention Care, and Reintegration. Each stage identifies the main actors involved, the ideal service components, users' experience, identified gaps, and potential explanatory factors. Critical pathway steps are highlighted in red. Source: Developed by CERRHUD based on the organisation's previous research.

### Context: country selection and study areas

The five countries were selected because of their participation in the *Ado Avance Ensemble* (AAE), a European Union-funded multi-country programme (2022–2025) coordinated by Rutgers in consortium with partner organisations in Benin, Burkina Faso, Cameroon, Côte d’Ivoire, and Togo and partners working at the regional level (IPAS and DKT) to improve sexual and reproductive health and rights outcomes for vulnerable adolescents, in particular out-of-school girls. This study was conducted within the broader AAE programme context, but as a distinct comparative qualitative research component commissioned by Rutgers and led by CERRHUD in close collaboration with Rutgers. AAE partner networks facilitated site selection, stakeholder identification, and access to some categories of participants. However, the research team retained responsibility for the study design, development of tools, analysis, interpretation, and manuscript preparation.

Within this programme context, country selection was guided by pragmatic considerations as the ongoing programme offered unique access to data and stakeholders and would ensure study findings could be acted upon within the programme context. However, the country selection also followed a purposive comparative logic: the five countries offered theoretically relevant variation in legal and policy trajectories regarding abortion after rape and incest.^17^ They represented contrasting configurations ranging from recent legal expansion (Benin) to partial alignment with procedural constraints (Burkina Faso), restrictive or only partially aligned legal frameworks (Cameroon), recent legislative reform (Côte d’Ivoire), and a relatively progressive but weakly operationalised framework (Togo). The comparative rationale was informed by variation across four dimensions: the degree of domestication of Maputo Protocol Article 14 (2)(c), the breadth of legal grounds for abortion after rape or incest, the existence or absence of implementing guidance, and the extent to which these provisions appeared operationalised in health and justice systems.^17^ Within each country, one rural and one urban area of the programme’s operational zones were selected for data collection to capture diverse perspectives across settings; these sites were not treated as separate units of analysis, but as subnational entry points for examining the implementation and lived experience of nationally defined legal and institutional frameworks (Table 1).

**Table 1.** Geographic distribution of study areas by country

Country	Study sites	Region / Department
Benin	Lokossa – Athiémé; Adjohoun – Bonou – Dangbo	Mono; Ouémé
Burkina-Faso	Centre; Centre-Ouest	Centre; Centre-Ouest
Côte d’Ivoire	Abidjan; Gontougou; San Pedro	Gontougou; San Pedro
Cameroon	Soa; Ebolowa	Centre; South
Togo	Savanes; Kara; Greater Lome	Savanes; Kara; Maritime

Note: This table presents the regions or departments included in the study areas for each country. Regional names follow national administrative divisions.

Source: Field data collected between February and June 2025.

### Author reflexivity

The research was conducted by five country-based young researchers, including three men (Benin, Burkina-Faso, and Cameroon) and two women (Côte d’Ivoire and Togo), supported by a multidisciplinary coordination team in Benin and the Netherlands. In line with both the study design and the AAE programme’s commitment to meaningful and inclusive youth participation, young researchers were deliberately engaged in the research design, tool development, data collection, country-level data analysis and dissemination. Recruitment criteria required prior experience in sexual and reproductive health research and in-depth interviewing, while experience with vulnerable populations and community engagement was considered an asset.

Given that all survivor participants were female, and that disclosure of sexual violence is shaped by gendered power relations, the team paid attention to how researchers’ gender, age, professional position, and institutional affiliation could influence rapport, disclosure, and interpretation. Interviews were conducted by the country-based young researchers. As each country team included a single young researcher responsible for data collection, male researchers were accompanied by a female colleague for interviews with survivors, as was the case in Benin. In some settings, access to survivor participants was facilitated by female focal points from local organisations, who helped with initial contact and safe interview arrangements. Interviews were conducted only after informed consent or assent procedures had been completed, and participants were reminded that they could decline to answer any question or stop the interview at any time.

A psychologist from Cameroon trained all young researchers in trauma-sensitive interviewing and remained available for counselling support when needed. These positional issues were discussed during research training, regular exchanges on operational and ethical challenges, and collaborative analysis sessions.

### Sampling

Purposive sampling was used to select informants with direct knowledge of the legal, institutional, community, and lived dimensions of access to abortion after rape or incest. Initial participants were identified through country-level stakeholder mapping and through the AAE programme partner network, which helped identify relevant institutions, organisations, and community entry points. The survivor’s pathway framework, which maps the sequence of steps and actors survivors encounter as they seek care and justice, also helped orient sampling by identifying the categories of actors involved at different stages of survivors’ trajectories to care and justice. For institutional and civil society actors, researchers approached ministries, health facilities, judicial and social service actors, NGOs, professional associations, and community leaders identified through this mapping. For survivors and relatives, access was facilitated where possible through trusted local organisations and service structures already in contact with potential participants. Snowball sampling was then used, where relevant, to identify additional key informants recommended by initial participants. Sampling focused on four categories of actors to optimise access to different perspectives:

- Institutional actors: representatives of the ministries of health, justice, and social action, representatives of the judicial system and law enforcement, representatives of social services, programme managers and decision-makers in reproductive health, health professionals trained and untrained in safe abortion procedures
- Civil society actors: non-governmental organisations (NGOs) working on sexual and reproductive rights, NGOs specialising in the fight against gender-based violence, professional health associations, youth activists and peer educators
- Community actors: community and religious leaders (male and female)
- Survivors and relatives: survivors of sexual violence aged 15 years and older, with priority given to survivors whose experiences involved a pregnancy resulting from rape or incest, as well as relatives of survivors (families, supporters). Some survivors who had not become pregnant were also included when their accounts helped document early barriers in disclosure, reporting, referral, and post-rape care.

Participants were informed about the research team’s affiliation, the study’s objectives related to understanding barriers to access to safe and legal abortion for sexual violence survivors, and that findings would be used to inform policy and programming. Researchers introduced themselves as members of a multi-country research team examining implementation of reproductive health laws. No prior relationships existed between researchers and participants, apart from initial contacts facilitated through the AAE programme partner network.

Exclusion criteria included inability to provide informed consent or assent, high risk of re-traumatisation (as assessed by the team and partners facilitating access), and situations of acute psychological distress requiring immediate care.

### Data collection

The overall study was conducted from November 2024 to June 2025. Preparatory activities, including documentary analysis, tool finalisation, and administrative procedures, began in November 2024. Primary qualitative data collection (interviews and focus group discussions) was then conducted progressively across the five countries, once the relevant national ethical approvals and administrative authorisations had been obtained. The data collection combined three complementary approaches: documentary analysis, semi-structured one-on-one interviews and focus group discussions:

- Documentary analysis: focused on core legal texts on abortion and SGBV (constitutions, penal codes, reproductive health laws), and public policy documents (national SGBV strategies, safe abortion guidelines). The analysis considered texts and documents published since 2000. The inquiries were carried out via official national legal databases, ministries of health and justice institutional sites, targeted research through specialised databases (Global Abortion Policies Database, Legal Atlas WHO) and reports from international organisations (Centre for Reproductive Rights, Guttmacher Institute). Because some legal and policy texts reviewed use the term “mother” defining legal indications for abortion, we preserved that terminology only when referring directly to the wording or logic of those provisions. Elsewhere in the manuscript, we use the terms “woman”, “pregnant woman”, or “survivor”, depending on context, to avoid unnecessarily normative legal language.
- Individual, semi-structured interviews: specific interview guides were developed for each category of actors. The interviews were conducted in French or local languages depending on the context (e.g. *Mooré* in Burkina Faso). All interviews were audio-recorded with participants’ permission and subsequently transcribed verbatim. In cases where participants declined audio recording (*n* = 10), detailed field notes were taken during and immediately after the interview.
- Focus group discussions: targeted community and religious leaders, including a deliberate focus on capturing the voices of women in positions of leadership. Each group discussion included 6–8 participants representing different religious denominations, ethnic groups, and geographic areas to ensure diverse representation.

A total of 164 interviews and 14 focus group discussions were conducted (Table 2). Data collection and preliminary analysis proceeded iteratively rather than sequentially: daily debriefings and ongoing review of field materials were used to assess whether additional interviews were still generating substantially new information within each actor category and country context.^18,19^ Saturation was therefore not treated as a single threshold reached simultaneously across all sites, but as a progressive judgment made by actor group and country team during data collection and revisited during early analytical phases.^18,19^ It was reached more quickly for some categories of participants than for others. The target for interviews with survivors and their relatives was deliberately modest, given anticipated access challenges, but even the lower targets (10 interviews per country) proved difficult to achieve. However, the interviews obtained were in general rich and provided deep insights, while key informants also recounted survivor stories, thereby complementing the first-hand data.

**Table 2.** Distribution of interviews conducted by country

Country	Institutional and civil society stakeholders	Focus groups (community actors)	Survivors and relatives	Total
Benin	24	3	7	34
Burkina-Faso	19	2	7	28
Côte d’Ivoire	36	3	2	41
Cameroon	17	3	10	30
Togo	35	3	7	45
Total	131	14	33	178

Note: This table summarises the number of individual and group interviews conducted with institutional actors, civil society representatives, community members, and survivors or their relatives in each country.

Source: Field data collected between February and June 2025.

### Data analysis

The analysis uses the conceptual frameworks presented above and adopted a four-phase, iterative, collaborative qualitative thematic analysis approach specifically designed to optimise analytical quality in transnational research contexts involving numerous researchers and progressive data availability.^20,21^ This analytical approach combined triangulation of data sources with cross-validation of interpretations as transcript material became available over time.^22^ Deductively, the analysis was anchored in the Berman framework, which guided the structuring of findings across macro, meso and micro levels. The survivor’s pathway framework was used complementarily to identify key stages in survivors’ trajectories, to support the organisation of barriers by pathway stage, and to interpret delays, detours, and ruptures in access to care and justice. Inductively, new codes were generated from debriefing notes, field materials, and progressively available transcript excerpts when participants’ accounts pointed to unanticipated barriers, practices, meanings, or interactions not fully captured by the initial analytical framework.^23^

- Phase 1: Real-time familiarisation and consolidation through daily online debriefings during country collection. These sessions addressed ethical and practical dilemmas, enabled immediate qualitative analysis, and identified emerging trends.^18^ At this stage, full verbatim transcripts were not yet available for all interviews and focus group discussions. A structured collaborative framework on the MURAL platform visually organised information by country and dimension, ensuring systematisation and traceability based on the Template analysis approach.^24^ This phase generated preliminary codes and early hypotheses for subsequent refinement.
- Phase 2: Generation of codes and consolidation by thematic analysis matrices: each country team developed consolidated analysis matrices, validated by the coordination team to identify convergences and contextual specificities. These matrices were informed by debriefing materials, field notes, and the first available transcript excerpts, and included illustrative citations organised around both deductive and inductively generated codes, while explicitly identifying the areas requiring further confirmation. Newly emerging inductive codes were discussed with the coordination team and were retained, merged, or reformulated as analysis progressed.^25^ At this stage, interpretations based primarily on debriefing materials and early transcript excerpts were treated as provisional, and the matrices were also used to flag areas of uncertainty, tension, or possible contradiction requiring later verification.
- Phase 3: Transnational co-validation and theme development: a three-day workshop with the full research team in Cotonou (Benin) enabled the validation of provisional codes and the identification of cross-cutting themes. At this stage, national teams worked with the country matrices and the transcript materials already available, using the Berman framework to compare interpretations across implementation levels and the survivor’s pathway framework to identify where along survivors’ trajectories key barriers, delays, and ruptures (that is, interruptions or breaks in survivors’ pathways to care and justice) were located. Consensus was sought on the interpretation and grouping of cross-cutting themes across countries, while country-specific codes were retained when they captured context-specific dynamics not shared across all settings.^25^
- Phase 4: Revision and refinement of the themes through full transcripts: complete transcripts were progressively integrated to validate and refine the themes and finalise the thematic analysis. Each newly completed transcript was reviewed against the established codes and themes to confirm consistency, identify any additional elements requiring refinement of the analysis, and actively examine whether the fuller transcript material contradicted, qualified, or nuanced interpretations developed from earlier debriefing notes, field notes, and partial transcript excerpts. Regular debriefings, coordination reviews, and collaborative analysis sessions also functioned as reflexive spaces in which the team questioned early assumptions, compared interpretations across countries, and considered how researcher position, field relationships, and progressive data availability could shape the reading of the material.

### Ethical considerations

The research obtained the required authorisations in each country and complied with international standards for research with vulnerable populations. The research protocol has been submitted and approved by the following National Ethics Committees: Local Ethics Committee for Biomedical Research of the University of Parakou in Benin (Ref: 820/2024/CLERB-UP/SP/R/SA, 21 November 2024); Ethics Committee for Health Research in Burkina Faso (deliberation No. 2024-11-353, 6 November 2024); National Committee for Ethics of Life Sciences and Health in Côte d’Ivoire (N/Ref: 000273, 16 December 2024); National Research Ethics Board for Human Health in Cameroon (N° 2025/02/1374/CE/CNERSH/SP, 6 March 2025); Bioethics Committee for Health Research in Togo (Opinion No. 0458/2024/CBRS, 5 December, 2024).

In each country, primary data collection involving interviews or focus group discussions began only after national ethical approval and the relevant administrative authorisations had been obtained, meaning that the data collection in Cameroon was delayed compared to other countries, and the project timelines were adjusted to accommodate the delays.

In addition, official authorisations were obtained from the Ministries of Health (and, where applicable, from the social services authorities) to conduct interviews within health and social care services.

Written informed consent (or informed assent and parental consent for minors) was obtained prior to each interview. Signed paper consent forms were scanned, uploaded to the study log, and checked by the research coordination team in Benin. Once this verification had been completed, the paper originals were destroyed in each country of data collection to reduce the risk of data breaches. To mitigate the risk of psychological distress, interview guides for survivors ensured interviewers emphasised and repeated – during the informed consent process and throughout the interviews – that survivors were free to stop the interview at any time without repercussions. Interview questions were carefully structured, beginning with general, community-level focused inquiries before moving on to personal questions, with instructions for interviewers to conduct regular comfort checks with participants. Interviewers received training to recognise signs of distress and to interrupt or redirect interviews when necessary, following established distress protocols. The protocol included instructions for referral to designated medical or psychological care providers as needed.

## Results

The results presented below represent the main barriers to effective access to safe and legal abortion care for survivors of sexual violence, as observed at three implementation levels of analysis. Barriers often overlap between the countries studied, while differences between countries are also indicated.

### Analysis of legal frameworks

The five countries studied show a variety of legal and political trajectories regarding access to a safe abortion, characterised by fluctuations between legislative advancement, gradual progress and periods of stagnation (Table 3). All five countries ratified the Maputo Protocol between 2003 and 2012 and legally authorise voluntary termination of pregnancy in cases of rape and/or incest. However, significant implementation gaps persist across all contexts, particularly regarding alignment between legal frameworks and operational protocols for providing abortion care and responding to gender-based violence. These gaps manifest differently across countries (Table 3; Supplementary Tables S1–S5).

**Table 3.** Legal and operational framework for voluntary termination of pregnancy and gender-based violence response in five West and Central African countries

Country	Maputo Protocol ratification	Primary VTP legislation (date)	Legally authorised VTP conditions	Key GBV operational texts (date)	Major implementation gaps identified
Benin	30 September 2005	Law N° 2021-12 (20 December 2021) amending the 2003 Law	Rape/Incest Threat to maternal health/life Severe fetal malformations Material, educational, professional, or moral distress	Decree N° 054 (22 June 2022): SRH service package including “safe abortion” GBV SOP (February 2023) Decree N°2023-151 (19 April 2023): operational conditions for VTP	The 2023 GBV SOP references the 2021 law but omits all mention of VTP access for sexual violence survivors, despite explicit legal authorisation for rape/incest. Indicates intersectoral coordination failure.
Burkina-Faso	9 June 2006	Penal Code (31 May 2018), Articles 513-13 and 513-14	Rape/incest Threat to maternal life Severe congenital malformations	SR Policies and Standards (May 2010) PAC/SAI Manual (August 2021) Code of Medical Ethics (7 February 2014): conscientious objection provisions	The 2021 PAC/SAI Manual imposes burdensome procedures: mandatory police report, and prosecutor approval and medical consultation within 14 weeks. No expedited mechanism for emergency cases
Côte d’Ivoire	Signed: 27 February 2004. Ratified: 5 October 2011	Law N°. 2024-358 (11 June 2024), Article 427	Rape and incest Threat to maternal mental and physical health Threat to maternal life	GBVIMS (April 2010) National GBV strategy (2014) Interministerial Circulars (2015-2016) Law 2021-894 on victim protection	Recent legislative evolution: 2019 Penal Code limited VTP to rape (excluding incest) and life-threatening conditions. The 2024 law significantly expands access by including incest and maternal mental health. Operational implementation remains to be evaluated
Cameroon	28 May 2009 Ratified: 2012	Revised Penal Code (12 July 2016), Article 339	Threat to maternal life (certification by authorised person) Rape (certification by republic ministry)	National GBV Protocol (October 2015) SRH/FP Standards (March 2018) SOGOC Referral Pathway	The 2018 SRH/FP Standards describe therapeutic abortion procedures for fetal malformations but completely omit procedures for rape victims, despite legal authorisation since 2016. Domestication of Maputo Protocol Article 14 ongoing (2025)
Togo	Signed: 30 December 2003. Ratified: 12 October 2005	Law N°. 2007-005 (10 January 2007) on reproductive health	Rape/incest Threat to maternal health High risk of severe fetal abnormality	National GBV Strategy (June 2012) Code of Medical Ethics (2013): conscientious objection provisions National GBV Protocol (September 2023)	2007 Law remains unimplemented after 18 years. The 2009 Public Health Code (Article 108) requires certification by three physicians, creating insurmountable barriers. The 2023 GBV Protocol does not mention legal abortion access for sexual violence survivors

Abbreviations: VTP = voluntary termination of pregnancy; GBV = gender-based violence; SRH = sexual and reproductive health; SOP = Standard Operating Procedures; PAC/SAI = post-abortion care/safe induced abortion; SRH/FP = sexual and reproductive health/family planning; SOGOC = Cameroonian Society of Gynecology and Obstetrics; GBVIMS = Gender-Based Violence Information Management System.

Source: Field data, November 2024 – June 2025. For complete legal architecture details for each country, see Supplementary materials – Tables S1 – S5.

Benin illustrates a pattern of legislative progression, transitioning from the 2003 reproductive health law, which permitted voluntary termination of pregnancy in cases involving threats to the life or health of the woman, rape or incest, or serious fetal malformation, to the comprehensive reform of 2021 (Supplementary Table S1). The latter expanded the legal grounds for abortion to include situations of material, educational, professional or moral distress, establishing differentiated gestational limits: 12 weeks of amenorrhea for cases of distress, extendable in cases of rape, incest or medical indications.

A distinctive characteristic of Benin’s legal framework lies in the incorporation of pregnancies resulting from sexual violence as a legitimate ground for abortion in national legislation as early as March 2003, predating the African Union’s adoption of the Protocol of the African Charter on Human and Peoples’ Rights on the Rights of Women in Africa (Maputo Protocol) in July 2003. However, the legislation remained without implementing regulations, creating a substantial void regarding operational guidance for an extended period. For the current law (2021), an implementing decree was published in 2023.

However, the the earlier Law No. 2011-26 on the prevention and repression of sex-based offences and the protection of women does not appear to provide explicit operational guidance on access to voluntary termination of pregnancy for survivors of sexual violence. Likewise, the Standard Operating Procedures (SOPs) for preventing and responding to gender-based violence, updated in 2023, refer to the 2021 reproductive health law but do not explicitly recognise abortion as a right for survivors of rape and incest, despite listing other specific health services such as testing for sexually transmitted infections (Supplementary Tables S1). This suggests that, despite recent legal progress on abortion, alignment between abortion law and the normative and operational texts governing the management of sexual violence remains incomplete. Furthermore, the amended reproductive health law from 2021 continues to reflect the prohibitive stance towards the promotion of abortion inherited from colonial-era legislation in its Article 19-1.

The case study from Benin sketches key challenges pertaining to legislative frameworks (including insufficient harmonisation between different laws and the persistent criminalisation of the promotion of abortion) and policy development (characterised by absence or delayed formulation) that may hamper the implementation of an existing abortion legislation. Similar challenges were found in the other countries.

Burkina Faso demonstrates a comparable pattern where progress has been achieved regarding the domestication of the Maputo Protocol article on abortion, but this coexists with insufficient enforcement mechanisms (Supplementary Table S2). The country allows abortion in circumstances of threat to maternal health, incurable serious fetal abnormalities (without gestational restrictions), and rape or incest (up to 14 weeks), contingent upon investigation by the prosecutor and a written confirmation of distress. Although this framework largely aligns with the Maputo Protocol, it fails to explicitly refer to mental health considerations as grounds for abortion. Legal inconsistencies persist, like in Benin, inadequate harmonisation exists between legal instruments and operational protocols, particularly those governing sexual violence and abortion services. Furthermore, as in Benin, Article 513-15 (Supplementary Table S2) criminalises “incitement to abortion” without a clear definition, while the prohibition of abortion advertising, inherited from the 1994 Public Health Code, perpetuates a climate of uncertainty and fear among civil society actors. In addition, Cameroon, for its part, maintains a particularly restrictive legal framework. Article 339 of the Penal Code, revised in 2016, allows abortion under only two circumstances: rape verified through certification from the public prosecutor's office, or when the pregnancy forms a serious threat to the woman's health (Supplementary Table S3). The 2018 Reproductive Health and Family Planning Norms and Standards introduced an additional ground by incorporating severe fetal malformations incompatible with life. The same document establishes gestational limits: before 12 weeks for cases of rape or medical reasons, and up to 28 weeks for life-threatening conditions or severe malformations. However, the provisions governing rape cases beyond 12 weeks remain ambiguous, as they appear to be subsumed under situations where the continuation of the pregnancy constitutes a vital risk for the mother, without clear delineation of what constitutes such risk. Despite the ratification of the Maputo Protocol in 2012, incest remains unrecognised as legal grounds for abortion, an inconsistency with international commitments. In addition, complex legal procedures and the absence of clear judicial guidelines undermine timely application of the provisions, exposing survivors to legal and administrative uncertainty. Civil society organisations, including the Cameroon National Association for Family Welfare (CAMNAFAW) and partners, are still actively advocating for the domestication of Article 14(2)(c) of the Maputo Protocol. They have had some success; at the end of the National Assembly’s session held on 27 March 2025, legislators urged the government to draft reproductive health legislation, to incorporate the provisions of Article 14 into national legislation and to revise the Penal Code to facilitate access to abortion in cases of rape, incest or danger to the mother. The proposed legislation is at the time of writing of this article in the process of being drafted.

Côte d’Ivoire illustrates a recent dynamic of reform (Supplementary Table S4). While the country ratified the Maputo Protocol in 2011, the termination of pregnancy was permitted solely to save the life of the woman in particularly urgent circumstances. In this case, the treating physician or surgeon was required to consult with two additional physicians. The 2019 revision of the Penal Code introduced rape as legal grounds for abortion, subsequently expanded by another amendment of the Penal Code in 2024 that significantly broadened access by including incest as well as considerations of the mental and physical health of the mother, while clarifying procedural requirements in cases of rape. This development positions Côte d’Ivoire as close to compliance with the Maputo Protocol, although it does not explicitly recognise life-threatening fetal conditions as legal grounds for abortion. The gradual adoption of Maputo Protocol article 14(2)(c) guidelines, reinforced by complementary legislation addressing gender-based violence, is promising. In particular, Law N°2021-893 amended the Penal Code by expanding the legal definition of rape and strengthening sanctions, while Law N° 2021-894 reinforced the protection of victims of domestic violence, rape, and sexual violence. However, these reforms do not in themselves provide operational guidance on access to legal abortion after sexual violence, and implementation guidelines are yet to be issued for the amended Penal Code articles on abortion.

Finally, Togo embodies a singular paradox (Supplementary Table S5). The 2007 reproductive health law, adopted after the ratification of the Maputo Protocol in 2005, demonstrates substantial alignment with the Maputo Protocol, permitting abortion in cases of rape, incest, threat to maternal health, or severe fetal malformation, with no gestational restrictions. However, this legislation remains largely inoperative eighteen years after its adoption due to a lack of operationalisation. Its non-implementation underscores the gap between statutory provisions and their translation into operational policies. These implementation gaps are mostly attributable to local resistance stemming from pervasive social tension surrounding abortion.

The detailed analysis of barriers identified at each stage is presented below, structured according to implementation level**.**

### Barriers to accessing safe abortion services

Table 4 provides a comprehensive overview of the barriers identified across all five countries, organised by stage of the survivor’s pathway, typology, and level of analysis (macro, meso, and micro). This pathway perspective makes it possible to identify not only the barriers themselves, but also the points at which survivors’ trajectories are interrupted, delayed, or redirected as they move from information and disclosure to reporting, medical and psychosocial care, legal or administrative authorisation, and abortion services. The following sections examine the most salient barriers at each level, beginning with macro-level structural impediments.

**Table 4.** Classification of barriers by stage of care pathway and level of intervention

Stage of the Pathway	Identified barriers	Typology	Level (Macro / Meso / Micro)	Countries concerned
Information & Awareness	Widespread lack of knowledge of abortion laws (even after reform)	Informational / Institutional	Macro & Meso	All countries
	Legal restrictions on abortion-related communication, promotion, or dissemination of abortion information, products, or methods	Legal	Macro	All countries
	Imposed silence, stigma, religious and family norms	Social / Cultural	Micro	All countries
Reporting	Delays in reporting due to fear, shame, threats, or ignorance of legal time limits	Social / Cultural	Micro	Cameroon, Burkina-Faso, Benin
	Late pregnancy discovery (after 3–4 months)	Institutional. / Individual	Micro	Cameroon, Benin
	Absence of simplified GBV reporting procedures	Institutional	Meso	Togo, Burkina-Faso
Medical Examination	Lack of trained physicians for medico-legal and safe abortion procedures	Institutional	Meso	All countries
	Insufficient medico-legal documentation and incomplete files	Institutional	Meso	Cameroon, Burkina-Faso, Benin
	Providers’ conscientious objection	Religious / Ethical	Micro	All countries
Psychosocial support	Scarcity of psychologists trained in GBV trauma	Institutional	Meso	Cameroon, Burkina-Faso, Benin
	Weak or absent structured psychosocial support	Institutional	Meso	All countries
	Family stigma (guilt, rejection)	Social / Cultural	Micro	Togo, Burkina Faso, Benin
Administrative / Legal Authorisation	Procedural complexity (collegial reviews, multiple opinions, judicial delays)	Institutional / Legal	Macro & Meso	Cameroon, Togo, Côte d’Ivoire, Burkina Faso
	Variability across jurisdictions or between urban / rural areas	Institutional	Meso	Togo, Burkina Faso, Benin
	Survivors are rarely involved in decision-making	Social / Institutional	Micro	Côte d’Ivoire, Benin
Access to abortion services	Faith-based facilities are systematically refusing abortion	Religious / Institutional	Meso & Micro	Cameroon, Côte d’Ivoire, Burkina-Faso
	Providers’ fear of prosecution due to lack of clear implementation texts	Legal	Macro	Togo, Burkina-Faso, Côte d’Ivoire
	Territorial inequalities (disadvantaged rural areas)	Economic / Geographic	Meso	All countries
	High costs despite legality	Economic	Macro & Micro	Cameroon, Burkina Faso, Côte d’Ivoire, Benin

Source: Field data collected between February and June 2025.

#### At the macro level

The obstacles that emerge at the macro level primarily relate to the legal and policy framework. They concern, among others, legislative ambiguity and the absence of implementing regulations, prohibitions on open communication regarding abortion, and cumbersome judicial and administrative procedures. These structural impediments, delineated below, influence institutional practices at the meso-level and increase susceptibility to service gaps at the micro level.

First, it is necessary to acknowledge the prevalence of legal ambiguities and the absence of implementing regulations. In Cote d’Ivoire, Togo and Cameroon, the lack of implementing regulations and standardised procedures generates uncertainty for magistrates and service providers throughout the implementation chain. According to the data, this ambiguity fosters divergent interpretations of legislation between judicial and healthcare actors and contributes to delays or obstructions in service access. The following observations illustrate this point:

“*[…] The [legal] texts provide that the public prosecutor must certify the materiality of rape in order to authorize abortion, […] but this creates a practical dilemma […]: Must the victim wait for the outcome of the trial before she can have an abortion? The procedures last 2 to 3 years, […] or even 5 to 6 years with appeals […].*” (Magistrate, Ebolowa, Cameroon)

“*We participate in many workshops where we think about how to accelerate the development of the implementation guidelines of the law on reproductive health to protect us in the exercise of our functions because the phenomenon is there. Per month, you can have ten cases of rape in consultation*.” (Gynaecologist, Healthcare facility, Lomé, Togo)

“*It is true that the law has evolved, but it must be disseminated and made understood. And above all, everything has to be really put in black and white, in documents that are easy to read, affordable and within the reach of the various stakeholders.*” (Senior health official of Abidjan, Côte d’Ivoire)

The existence of uncertainty surrounding the implementing guidelines undermines the legal certainty of actors and reinforces the hesitation of providers, creating barriers at the stage of legal recognition of survivors’ rights.

Second, legislation prohibiting unrestricted communication about abortion by prohibiting its “promotion” exists in all the countries in this study. This prevents public dissemination of information on rights and procedures. These prohibitions, far from being merely theoretical, influence the conduct of healthcare professionals and civil society organisations. For example, Côte d’Ivoire's Penal Code (Articles 426 and 428) explicitly prohibits the advertising, sale, or distribution of information related to incitement to abortion. In Benin, despite progressive legislation, Article 19-1 of Law No. 2021-12 explicitly prohibits all propaganda related to safe abortion and establishes criminal sanctions for violations. While these provisions primarily target promotion and incitement rather than dissemination of information, they generate uncertainty and apprehension among civil society organisations and healthcare professionals about communication on this sensitive subject. These restrictions also contribute to a significant lack of public awareness (including survivors and their relatives) concerning laws and procedures related to abortion and sexual violence.

Third, the data show that effective access to safe abortion is contingent on judicial or administrative authorisation in several countries. The absence of statutory deadlines for the authorities to issue these authorisations, procedural delays and unclear or non-existent procedures constitute significant obstacles. The following statements testify to this phenomenon:

*“In the national protocol for the care of victims of GBV […], the conduct to be taken when the girl becomes pregnant is not mentioned. We don't know the procedure to follow, […], so when this happens, we inform the judicial police officer and wait for the prosecutor's authorization […]. Out of three requests already made, we had received no response, and some girls had to keep the pregnancy and others found other unsafe ways to evacuate the pregnancy.”* (Key informant of a GBV victim care centre, Lomé, Togo)

*“Now, the question arises as to the applicability of the texts. As far as the rape case is concerned, a judicial investigation must be opened. This is really the opening of the investigation. In this case, questions of delay will arise since the pregnancy lasts nine months.”* (Member of an association, Abidjan, Côte d’Ivoire)

These procedural burdens, which are present in all countries except for Benin, reflect a disconnect between legal recognition of the right to safe abortion and its practical implementation. In Benin, since the 2021 law, opening a judicial investigation is no longer a requirement for a survivor of rape or incest to access safe abortion services.

#### At the meso level

The obstacles identified at the meso level primarily concern the organisation and practices of healthcare structures, social services, judicial institutions and intermediary actors. The findings reveal divergent institutional practices.

First, the data identify a difference in the adoption strategy by non-governmental organisations (NGOs) and private institutions. In several countries, NGOs and private clinics serve as alternatives to the public system. Some develop coping strategies (such as interpreting rape as constituting a health risk, whether physical or mental) or refer to online referral systems to access abortion medication. However, the operational scope of NGOs and private clinics remains severely limited due to restrictive legislation and inadequate funding. These factors result in limited coverage areas and increased risk of institutional marginalisation. In addition, the NGO sector is polarised: some faith-based NGOs explicitly refuse to communicate about abortion and only offer support for childbirth, despite legal permission for termination. As expressed by an interviewee in Togo: “*We can't talk to a woman about abortion. Abortion is prohibited in Togo”* (Interview with a leader of a civil society organisation, Togo)*.* This attitude results in a significant lack of information and limits survivors’ access to their rights.

Second, public healthcare facilities are not adequately prepared to provide safe abortion care. Healthcare structures frequently report insufficient information regarding guidelines, inadequately trained staff, or unavailable appropriate medications. Moreover, many institutions remain unprepared regarding values and attitudes. Even after a victim has obtained the requisite legal authorisation for the safe termination of pregnancy, she may encounter challenges in receiving care, as illustrated by this example from Burkina Faso:

“*I met two women who complained. […] [One of them] went from one structure to another, like a ping-pong ball. She was told: ‘No, we don't do that here, we have to go elsewhere,’ and in the new structure, she was sent away again. The doctor who was present played the game to wait for his colleague from the next shift, the midwives also said that it is not up to them to start the protocol and so on. […] one of the women has been going to three structures without a solution. This problem persists.*” (Key informant of a professional association, Ouagadougou, Burkina Faso)

Third, insufficient coordination among healthcare, justice and security, and social affairs structures impede integrated care for survivors. Each institution operates according to its own rules, without a common protocol, resulting in fragmented care, inadequate referrals from one structure to another, and institutional displacement. As these participants emphasise:

“*For example, when it comes to sexual violence such as rape […] When we go to certain centres for the care of victims of sexual and gender-based violence, the people who welcome us will tell you that no, you should first go to the police to denounce and it is to the police that we will give you a document to come and do tests, kits, medical care. You go to the police; they will ask you where the [medical] document is that proves that you have been a victim of rape. So finally, people wonder if I should go to the police first or to the hospital first.*” (Head of a local NGO, Lomé, Togo)

A social worker in Benin described a procedural bottleneck in cases involving child survivors of rape: police and judicial handling were repeatedly delayed because the required medical certificates were not finalised by the medical staff when the survivors and the alleged perpetrator were brought before the relevant authorities on several occasions.

*“[…]the child survivors were first brought in on Monday. Because the medical certificate was still missing, police custody had to be extended. The perpetrator was presented again on Thursday, and once more the child survivors were brought in without the certificate. […] That was yesterday and since then, we still haven't received the medical certificates.”* (Social worker, Lokossa, Benin)

The lack of coordination prolongs the time it takes to receive care and justice, multiplies procedural breaking points, and increases the risk of survivors disengaging from the justice- and care-seeking process, as will be demonstrated below.

### At the micro level: barriers and ruptures along the survivor’s pathway

At the micro level, barriers directly affect the individual actors involved in both the supply and demand of safe abortion services. This includes providers, survivors, and their immediate social environment, including families and communities. Analysed through the survivor pathway, these obstacles appear across the successive stages of the disclosure, reporting, medico-legal and psychosocial care, legal or administrative authorisation, and access to abortion services. Rather than occurring in isolation, they accumulate along the pathway and compound the constraints already imposed at the macro and meso levels, resulting in repeated delays, detours, or definitive ruptures in access to care and justice.

First, the data demonstrate that insufficient awareness of the legal framework constitutes a major obstacle. Many survivors remain unaware that they have a legal right to safe termination of pregnancy under specific circumstances (rape, incest, life-threatening conditions). This lack of information is partly the result of the absence of institutional communication and the criminalisation of advertising or incitement to abortion in all contexts. The following assertions illustrate this phenomenon:

“*You can raise awareness about sexual violence but in all the services available, you can't afford to talk about abortion – people will say that you are advocating for abortion. We had already received threats in this regard.*” (Activist in a local NGO, Lomé, Togo)

“*The law on abortion does not allow all issues related to abortion to be addressed as we hear it in the media […] I personally think that the government has put a clause in the law where today the media cannot talk about this law as they want.*” (Journalist, Cotonou, Benin)

“*[…] many girls who have been raped, they themselves do not know what path to follow in order to have access to abortion services. And most of the time they find themselves getting clandestine abortions because they don't have the information […]*.” (Leader Youth Organisation, Soa, Cameroon)

Some survivors report discovering these provisions only after exceeding the legal deadlines, and also that their relatives remain uninformed about these rights. Survivors say that they were never informed of this option when reporting to social services, healthcare professionals or the police immediately after the rape, or subsequently when seeking assistance after discovering that they were already pregnant. As related in this account:

“*I wanted to have an abortion, I couldn't keep my uncle's child, no I didn't want the pregnancy (…) No, at the time I didn't know that when you are a victim of rape, you could have an abortion, I thought that abortion was forbidden, that's why I went back to the village to take advice from my peers on how to have an abortion.*” (Survivor, Kara, Togo)

“*I didn't know that in Burkina Faso the law allows the abortion of a pregnancy resulting from rape or incest. From the moment you find out you’re pregnant, you could interrupt, if I had known and could have interrupted, I wouldn’t have suffered so much.*” (Survivor in Ouagadougou, Burkina Faso)

This lack of information leads to avoidance of the legal system and encourages the use of clandestine and unsafe alternatives.

Other obstacles include social and religious stigmatisation. This phenomenon is pervasive throughout the survivors’ testimonies. Social and religious frameworks frequently lead to moral condemnation of the request for an abortion. Families, communities and occasionally religious leaders respond by rejecting, blaming or isolating survivors. Reports indicate that survivors have been compelled by those close to them or have been advised to continue the pregnancy despite the availability of legal options.

“*As pastors confronted with this case, we prayed that the young girl would give birth in the right conditions. For me, conceiving your child in a rape is not bad, the child has nothing to do with it. The church does not encourage abortion.*” (Religious leader, Ebolowa, Cameroon)

“*So, it’s complicated. The girls are afraid to talk about it. And then often, the threats, the pressure from the family, it’s complicated. So as a result, they look after children when they don’t necessarily want to. And it’s complicated.*” (Blogger, Abidjan, Côte d’Ivoire)

Others point to the fear of social exclusion, which prevents them from turning to institutions for help. Several testimonies reveal severe forms of suffering and exclusion faced by women who report perpetrators:

“*After the first episode, I had horrible months in the neighbourhood. The man’s family came to insult me, threatening me with death until my uncle took me out of the house. I was perceived as the devil. It traumatized me.*” (Rape survivor, Kpalimé, Togo)

“*[…] the poor woman who identified her executioner was insulted by the community. She was taken for a bad woman, especially since she had even gone to court with him. When she came back, she really couldn’t even go out in the neighbourhood.*” (Member of an NGO, Abidjan, Côte d’Ivoire)

Severe forms of victim-blaming and inequitable gender norms that protect perpetrators rather than survivors reinforce the silence surrounding sexual violence and impede access to safe services. Some families also impose reconciliation with the perpetrator of the rape, sometimes orchestrated by community leaders or institutional agents, whether in the healthcare sector or judicial system. The logic of preserving family honour obstructs institutional pathways and delays medical care, sometimes rendering abortion legally impossible due to missed deadlines.

Fourth, conscientious objection (the option offered to doctors to refuse to perform an abortion on grounds of conscience or religious belief), although situated at the intersection between institutional and individual levels, manifests at the micro level. The conscientious objection clause existing in each country either in the legal articles on abortion or in the medical ethics codes, complicates adopting legislation in public institutions and undermines equitable access to services. While conscientious objection is infrequently invoked by providers (when used as grounds for service refusal, the law requires referral to another provider), it is employed informally, often expressed through negligent behaviour, which discourages survivors and may lead them to pursue alternative, frequently dangerous options.

“*There are colleagues who do not perform abortions. According to the law, a doctor is not obliged to perform an abortion. So, if it is against his convictions, he can try not to perform the abortion.*” (Doctor, Abidjan, Côte d’Ivoire)

“*It’s true that there is a law. But people have their prejudices. They are still there. So, this makes that even the victims who do come depend on the prejudices of the one who receives her, who can convince the victim about his own beliefs. So, these are also traps we see often.*” (Healthcare Provider, Lokossa, Benin)

Due to the various types of barriers that manifest at the micro-level, survivors encounter definitive disruptions in their pathways to care and justice, sometimes after already having navigated several administrative and judicial stages. This situation increases their feeling of abandonment and exposes women to unsafe abortions. This occurs particularly among survivors for whom informational and social obstacles are so substantial that they pursue local and informal solutions to their unintended pregnancies.

## Discussion

Analysis across the five countries shows that survivors of rape and incest encounter systemic barriers to accessing safe and legal abortion at three levels: macro, meso, and micro. These barriers are consistent with observations in comparable contexts, while also providing new insights into how legal exceptions for sexual violence are undermined in practice.

Consistent with other studies, our research demonstrates that ambiguous or restrictive legal frameworks limit effective access to safe and legal abortion after rape or incest, even when such access is formally permitted under specific legal exceptions. For example, Bankole et al. note that 92% of women of reproductive age in sub-Saharan Africa live in countries with highly restrictive or unclear legislation, contributing to legal uncertainty for both providers and users.^6^ Violence is a major cause of unintended pregnancies in the region, putting survivors at an elevated risk of unsafe abortions.^2,26^ Estimations based on data from 2015 to 2019 demonstrated that most abortions in sub-Saharan Africa were performed unsafely, with 77% being conducted using unsafe methods and/or by untrained providers, compared to the global average of 45%.^6^ Specifically, the Central African sub-region had the highest case fatality rate globally (260 deaths per 100,000 abortions), followed by West Africa (225 deaths per 100,000 abortions), according to the same 2019 estimates. In the whole of sub-Saharan Africa, this meant an estimated 15,000 preventable deaths related to unsafe abortions occurred annually.^6^ Although abortion-related mortality has decreased over time in the region, due to increased access to and quality of post-abortion care and increased (often clandestine) use of misoprostol, the burden of unsafe abortion remains disproportionately high in sub-Saharan Africa, with the highest abortion case-fatality rate globally.^6^ Similarly, qualitative research in Nigeria identifies legal and operational barriers as primary drivers of persistent unsafe abortion rates.^27^ This study confirms that restrictive or ambiguous legal frameworks constitute major obstacles in survivors’ pathways to health and rights, despite existing exceptions for rape, incest, or life-threatening circumstances.

Beyond the legal ambiguity itself, our findings show that survivors’ access is frequently blocked by procedural requirements, including authorisation by prosecutors, judges, doctors, or parents, as well as mandatory police reports. In practice, these requirements transform legal guarantees into barriers to care. International guidelines recommend eliminating such procedural barriers that are incompatible with human rights and standards for quality of care.^28–30^ In addition, in line with other scholarly work, our research shows that criminalisation and regulatory statutes targeting abortion-related advertising, incitement or disseminating information, fuel fear of prosecution, reduce the visibility of services, and discourage staff from informing or referring patients.^31,32^

Another important finding is that ethical positions adopted by public health structures and social services critically influence survivors’ outcomes. These positions, including institutional and informal conscientious objection, can explicitly or implicitly constrain the availability of abortion services.^33^ Merner et al., for example, report that faith-based health facilities frequently impose institutional policies more restrictive than national law, limiting access even where abortion is legally permitted.^34^

Some faith-based health facilities and faith-based NGOs adopt stances that go beyond refusing pregnancy termination services: they actively promote maternal resilience ideologies, encouraging survivors to continue pregnancies to term. This orientation is reflected in spiritual or psychological support programmes aimed at fostering acceptance of motherhood, even when resulting from rape or incest. They thus contribute to the dissemination of a normative discourse that values the continuation of pregnancy resulting from sexual violence.^35^ The fragmented care landscape, compounded by public health system limitations and the conscientious objection practices, produces highly unpredictable access to care.^33^

Overall, the findings emphasise that access to quality, safe and supportive services for survivors is not only unpredictable from one encounter to another, but also unequally distributed across settings. In practice, access varies according to the type of institution encountered, territorial location, and the availability of trained and willing actors able to provide or facilitate care. The findings also show that NGOs, such as family planning associations, may partially compensate for public sector gaps by receiving referrals, facilitating access to willing providers, or interpreting rape as a threat to physical or mental health within existing legal frameworks. However, their scope remains limited by restrictive legislation, inadequate funding, and uneven territorial coverage. As McGinn and Casey indicate, while these initiatives provide value, they cannot substitute for robust institutional regulation.^36^

In addition, this study demonstrates that social and religious norms continue to heavily influence survivors’ trajectories after sexual violence, consistent with findings by Jim et al. in South Africa.^37^ In some cases, these norms go beyond discouraging abortion and actively frame the continuation of pregnancy after rape as morally preferable, thereby minimising the violence experienced by survivors and legitimising pressure to continue the pregnancy. Similarly, qualitative research in Kenya and India has revealed that fear of family or community judgment compels women to conceal their situation, delaying or preventing access to safe services.^38^ At the micro level, limited knowledge of legal rights constitutes another barrier. This information gap limits access to voluntary termination of pregnancy even with permissive legal frameworks, reflecting findings from studies conducted in East Africa. For example, research in Ethiopia has found that while the majority of women are aware of the existence of permissive laws, their understanding of procedural modalities remains limited, with prevailing conservative social attitudes exacerbating the problem of access.^39,40^ Consistent with Ouédraogo et al.,^41^ this study demonstrates that decisions to terminate a pregnancy frequently involve family negotiations or social constraints that diminish survivor autonomy. Young women often encounter emotional or financial pressure from family members and must negotiate their choices in unsupportive environments.^41^

Finally, the results suggest that barriers to safe and legal abortion after rape or incest operate through systematic interaction between the macro, meso, and micro levels, creating cumulative dynamics that divert survivors towards unsafe abortions or forced continuation of pregnancy. Both come with additional stigmatisation and long-term consequences. Legal restrictions and procedural ambiguity at the macro level constrain protocol dissemination at the meso level, resulting in inconsistent institutional decision-making regarding abortion access for sexual violence survivors. This fragmentation produces what researchers term an “institutional lottery”, where access depends primarily on which facility or authority a survivor encounters, rather than her legal situation.^42^

These cross-level dynamics are ultimately experienced by survivors as ruptures in their pathways to care and justice. Even where legal grounds for abortion exist, survivors may move from one institution to another without clear information, encounter informal or formal refusals, or abandon the process because of delays, stigma, fear, or lack of support. The analytical point is therefore not only that micro-level barriers exist, but that macro-level legal ambiguity and meso-level institutional fragmentation are ultimately lived by survivors as discontinuous and unequal pathways to care and justice. This cumulative attrition helps explain why legal entitlement does not translate into effective access and why coordinated action across legal, organisational and community levels remains necessary.^5^

### Strengths and limitations

This research has three key strengths. First is its comparative multi-country design, covering five diverse national contexts in West and Central Africa. This diversity makes it possible to identify both universal barriers and context-specific factors. Second, the service pathway analysis examines concrete journeys of survivors and their relatives, moving beyond approaches that focus solely on legal reforms or institutional frameworks to systematically document where delays, detours, and ruptures occur along trajectories to care and justice, especially at the micro level. Finally, the multilevel analytical framework (macro, meso, micro) illuminates dynamic interactions across normative, organisational and individual dimensions, providing systemic insights that advance scientific discourse on access to safe abortion.

Several limitations warrant consideration. The stigma surrounding abortion and its criminalisation may have influenced participant responses, potentially resulting in under-reporting or self-censorship. Second, while underlying dynamics identified in this study may be relevant for countries with similar institutional or legal environments, the particularities of these five countries preclude wholesale generalisation to the entire West and Central African region, or to other African contexts. In addition, data availability was not uniform across countries. Direct survivor and family accounts were more limited in some settings than in others, and legal or operational documents were not equally accessible or available in all countries. Documentation of some implementation processes, particularly judicial procedures, referral practices, and frontline decision-making, was also richer in some contexts than in others. As a result, some contributing factors, especially those related to pathway ruptures, operational guidance, and intersectoral coordination, were documented more unevenly across the five countries.

## Conclusion

This study, conducted in five countries in West and Central Africa, demonstrates that survivors of sexual violence experience restricted access to safe and legal abortion despite legal provisions permitting abortion after rape and incest. The analysis reveals interconnected obstacles at three levels: at the macro level, legal ambiguities and procedural constraints impede implementation (although at this level a lot of progress has been made in Benin recently); at the meso level, fragmented organisational systems and restrictive internal policies reduce the availability of services; while at the micro level, stigmatisation, insufficient awareness of rights, and social pressures undermine survivors’ decision-making autonomy. These obstacles mutually reinforce one another, generating a cumulative dynamic that accounts for the persistent reliance on unsafe practices. By documenting these mechanisms via a comparative, multi-level approach and by showing how they translate into concrete pathway ruptures for survivors, the study underscores the need for coordinated interventions that simultaneously integrate legal, institutional and social dimensions to transform the legal codification of rights and provide effective access.

## Supplementary Material

Supplemental Tables S1-S5

## Data Availability

The data underlying this article will be shared on reasonable request to the corresponding author.

## Provenance statement

This article was not commissioned and went through external peer review.
