# Supplementary material for: Safe and legal abortion access for survivors of rape and incest in West and Central Africa: a comparative case study analysis of key barriers
Source: Sex Reprod Health Matters. 2026 Sep 23;33(1):2735510. doi: 10.1080/26410397.2026.2735510 (PMC13700467; doi:10.1080/26410397.2026.2735510)
Supplement: Supplementary file 1 [file ZRHM_A_2735510_SM8328.docx]

**SUPPLEMENTARY MATERIAL:**

Detailed legal and policy frameworks for reproductive health and gender-based violence in African countries (Benin, Burkina-Faso, Cameroon, Côte d’Ivoire, Togo).

This supplementary material provides chronological documentation of constitutional, legislative, international, operational, and institutional texts governing access to voluntary termination of pregnancy (VTP) and management of gender-based violence (GBV) in Benin, Burkina Faso, Cameroon, Côte d'Ivoire, and Togo. Each table presents texts categorized by type (Constitutional, international, legislation, operational, institutional, services) with adoption dates and key content describing provisions, gaps, and implementation challenges.

**Supplementary Table S1**

Legal and policy framework for reproductive health and gender-based violence in Benin

| **Category** | **Text** | **Date** | **Key content** |
| --- | --- | --- | --- |
| Constitutional | Constitution | 1990 | The constitution enshrines the principle of equality between men and women |
| International | Convention on the Elimination of All Forms of Discrimination against (CEDAW) | 1992 | The country ratifies the Convention on the Elimination of all forms of discrimination against women |
| Legislation | Law N°. 2003-04 | 3 March 2003 | The law on sexual and reproductive health authorizes VTP under three conditions |
| International | Maputo Protocol | 30 September 2005 | Benin ratifies the Maputo Protocol on the Rights of Women in Africa including Article 14(2)(c) on abortion |
| Legislation | Law N°. 2011-26 on the prevention and repression of violence against women | 9 January 2012 | This law establishes a comprehensive framework for preventing and punishing violence against women. The law does not reference the right to VTP following sexual violence |
| Legislation | Penal Code | 2018 | The new Penal Code incorporates provisions relating to abortion in Articles 513-518, and rape and sexual assault (Article 553-557) |
| Legislation | Law N°. 2021-12 amending and supplementing Law N° 2003-04 of 3 March 2003 on sexual and reproductive health | 20 December 2021 | This law amends the 2003 legislation by authorizing VTP in four distinct situations, including material, educational, professional or moral distress. Offering services to perform VTP in public, in meetings or in writing, in punishable under Article 519 of the Penal Code, as is the promotion of means objects and procedures relating to VTP. |
| Operational | Decree N°. 054/MS/DC/SGM/C/SA of 23 June 2022 establishing the service package for sexual and reproductive health | 22 June 2022 | The decree explicitly includes “safe abortion services” in the package of services relating to sexual and reproductive health |
| Operational | Standard Operating Procedures (SOP) for the prevention and response to GBV | February 2023 | This protocol standardizes procedures for the management of gender-based violence (updated since 2014). Despite reference to the 2021 law on reproductive health, it does not mention access to VTP for survivors of sexual violence, constituting a major gap in intersectoral coordination |
| Operational | Decree N° 2023-151 establishing the conditions for voluntary termination of pregnancy | 19 April 2023 | This decree establishes the operational conditions for VTP with operational definitions, including conscientious objection for healthcare professionals |
| National | National Council for the Fight against Violence against Women (CNLVFF) | Since 2018 | This body coordinates national actions against violence against women |
| Services | Integrated Care Centers (CIPEC) | Ongoing | These Centers provide holistic care for victims at the departmental level |

Source: Field date, November 2024 – June 2025

**Supplementary Table S2**

Legal and policy framework for reproductive health and gender-based violence in Burkina Faso

| **Category** | **Text** | **Date** | **Key content** |
| --- | --- | --- | --- |
| Constitutional | Constitution | 1991 | The Constitution guarantees the fundamental rights of all citizens |
| International | CEDAW | 1984 | The country ratifies the Convention on the Elimination of All Forms of Discrimination against Women. |
| Legislation | Public Health Code | 13 May 1994 | Article 89 prohibits advertising, sale and information relating to abortifacients, as well as incitement to abortion in public places. This prohibition on promotion can be misunderstood and give rise to an erroneous fear of a total ban on information about legal abortion |
| Legislation | Penal Code | 13 November 1996 | This revision expands authorization for abortion for rape, incest and congenital malformations (previously limited to saving maternal life) |
| Legislation | Law N°. 049-2005/1N of 22 December 2005 on Reproductive Health | 22 December 2005 | This law establishes the general framework for reproductive health in Burkina-Faso |
| International | Maputo Protocol | 9 June 2006 | Burkina Faso ratifies the Maputo Protocol on the Rights of Women in Africa, including Article 14(2)(c) on abortion |
| Operational | Policies and Standards on Reproductive Health | May 2010 | This document defines the main guidelines for reproductive health. It confirms existing laws (three conditions) and emphasizes the prevention of clandestine abortions among young people and post-abortion care |
| Legislation | Decree N°. 2014-048/PRES/PM/MS on the Code of Medical Ethics | 7 February 2014 | Article 117 establishes the conditions for physicians to perform therapeutic abortion, as well as the modalities for exercising medical conscientious objection |
| Legislation | Law 061-2015/CNT on violence against Women and Girls and Protection of Victims | 6 September 2015 | The law recognizes and criminalizes violence against women and girls, including cultural, economic, moral, psychological, property-related, physical and sexual violence, but fails to mention the right to VTP for victims of sexual violence |
| Legislation | Penal Code | 31 May 2018 | Articles 513-13 and 513-14 authorize safe induced abortion under three specific conditions; Articles 513-14 criminalizes incitement to abortion by any means of distribution or advertising |
| Operational | Post-Abortion Care and Safe Induced Abortion Care Manual authorized by Law (PAC/SAI Manual) | August 2021 | This manual guides providers on post-abortion care a legal safe induced abortion, specifying that in cases of rape/incest, a police report must be filed, Prosecutor approval obtained, then medical consultation within 14 weeks. Protocols: misoprostol 400 μg SL/3h or surgery. |
| Coordination | Family Health Directorate (DSF) | Ongoing | The Family Health Directorate assumes the role of technical coordination for SRH activities |
| Services | Specialized GBV Care Center (CPEC-VBG) | Ongoing | This specialized center provides psychosocial support for victims of violence |

Source: Field date, November 2024 – June 2025

**Supplementary Table S3**

Legal and policy framework for reproductive health and gender-based violence in Cameroon

| **Category** | **Text** | **Date** | **Key content** |
| --- | --- | --- | --- |
| Constitutional | Constitution | 1972/1996/2008 | The Constitution declares that all human beings, regardless of race, religion, sex or belief, have inalienable rights |
| International | Decree N°. 83-166 on the Code of Medical Ethics | 12 April 1983 | This decree prohibits all abortive maneuvers |
| Legislation | Law N°. 90-035 on the pharmacy profession | 10 August 1990 | Article 75 prohibits the sale, distribution or promotion of abortifacient products, as well as speeches or written documents inciting abortion in public places. |
| International | CEDAW | 23 August 1994 | The country ratifies the Convention on the Elimination of Discrimination against Women |
| International | Maputo Protocol | 28 May 2009 | Cameroon ratifies the Maputo Protocol on the Rights of Women in Africa, including Article 14(2)(c) on abortion. |
| Operational | Strategic Plan for Reproductive Health (2014-2020), Strategic Plan for Adolescent Health (2015-2019), National Multisectoral Program to Combat Maternal Mortality (2014-2020) | 2014 | Various national plans relating to reproductive health have been implemented, GBV is a recurring theme, but no document explicitly refers to therapeutic abortion services |
| Operational | National GBV Protocol | October 2015 | This protocol, mentioned in field data, organizes the care of victims of gender-based violence. It is not specified whether it explicitly mentions legal abortion services in case of rape or incest |
| Legislation | Revised Penal Code | 12 July 2016 | Article 339 authorizes therapeutic abortion in two specific cases: il the mother’s life is in danger (justified by an authorized person) or if the pregnancy results from rape (after certification from the ministry of the republic) |
| Operational | SRH/FP Standards | March 2018 | This document defines standards for sexual and reproductive health and family planning, including severe embryonic and fetal malformations that impact the mother’s mental health. It describes the steps before therapeutic abortion but omits those for rape victims |
| Operational | SOGOC Referral Pathway National Strategic | Ongoing | SOGOC develops a “Referral Pathway” to guide survivors. |
| Operational | National Strategic Plan for Reproductive Health (2024-2030) | 13 March 2025 | Plan not yet available at the time of this study |
| Event | Plenary session on the domestication for Article 14 of the Maputo Protocol | 27 March 2025 | Among the flagship measures adopted are the drafting of specific legislation on Reproductive Health and revision of the Penal Code concerning medicalized abortion |

Source: Field date, November 2024 – June 2025

**Supplementary Table S4**

Legal and policy framework for reproductive health and gender-based violence in Côte d’Ivoire

| **Category** | **Text** | **Date** | **Key content** |
| --- | --- | --- | --- |
| Constitutional | Constitution | 1960, 2000, 2016 | The Constitution affirmed the equality of all citizens before the law, without distinction of origin, race, ethnicity, sex or religion |
| Legislation | Penal Code | 31 July 1981 | Abortion is totally prohibited and punished in Côte d’Ivoire, thus introducing the first criminalization of rape in Ivorian law. |
| International | CEDAW | 20 December 1995 | The country ratifies the Convention on the Elimination of Discrimination against Women |
| International | Maputo Protocol | 27 February 2004 | The country signs the Maputo Protocol on the Rights of Women in Africa, Including Article 14(2)(c) on abortion. |
| Operational | Gender-Based Violence Information Management System (GBVIMS) | April 2010 | Introduction of a system for collecting, storing and analysing data on GBV, with the primary objective of continuously providing reliable data |
| International | Maputo Protocol | 5 October 2011 | The country ratifies the Maputo Protocol |
| Operational | National GBV Strategy | 2014 | This strategy organizes public intervention on the issue of GBV according to five areas of intervention |
| Operational | Circular N°. 005 on the reception in Judicial Police services of complaints forms victims of physical assault | 18 March 2015 | This circular facilitates the first level of GBV reporting |
| Operational | Circular N°. 15/MJ/CAB on the reception of rape complaints | 13 July 2016 | This circular specifies the elements of rape and reminds police officers that they must receive the victim’s complaint, with or without a medical certificate |
| Operational | Interministerial Circular N°. 016/MJ/MEMIS/MPRD on the reception of complaints following GBV | 4 August 2016 | This circular recognizes the lack of relevance of the medical certificate when receiving complaints in cases of GBV. |
| Legislation | Penal Code | 10 July 2019 | This code establishes a restrictive framework for abortion, authorizing it only to save the mother’s life or in case of rape (excluding incest). Healthcare professionals who perform unauthorized abortions are punished |
| Legislation | Law 2021-893 amending the Penal Code | 2021 | This law expands the definition of rape and sanctions |
| Legislation | Law 2021-894 on the protection of victims of domestic violence, rape and sexual violence. | 2021 | This law strengthens the legal arsenal to better protect victims and punish perpetrators of such violence |
| Legislation | Law N°. 2024-358 | 11 June 2024 | Article 427 expands access to abortion in three cases: rape and incest, maternal mental and physical health, and maternal life. |
| Institutional | National Council for the Fight against Gender-Based Violence (CNLVBG) | Since 2000 | The national Council for the Fight against GBV coordinates national actions |
| Institutional | Directorate for the Promotion of Gender Equity (DEPG) | Since 2006 | The directorate aims to create a national body to promote gender equality and combat discrimination |
| Institutional | National Women’s Council | Since 2015 | This coordination body oversees policies relating to women |
| Institutional | National Observatory for Equity and Gender (ONEG) | Since 2019 | ONEG monitors policies |

Source: Field date, November 2024 – June 2025

**Supplementary Table S5**

Legal and policy framework for reproductive health and gender-based violence in Togo

| **Category** | **Text** | **Date** | **Key content** |
| --- | --- | --- | --- |
| Constitutional | Constitution | 1960, 1963, 1979, 1992, 2024 | The Togolese Republic ensures equality before the law for all its citizens; Article 11 of the 1992 Constitution stipulates that “men and women are equal before the law” |
| Legislation | Penal Code | 1980 | The French low of 1920, which limited abortion to cases of danger to the woman’s life, remained in force until 2006 |
| International | CEDAW | 1983 | The country ratifies the Convention on the Elimination of all Forms of Discrimination against Women |
| Legislation | Law N°. 84-14 on the protection of learners | 1984 | This law condemns anyone who would contribute to causing an abortion in a girl enrolled in a school or vocational training center |
| International | Maputo Protocol | 30 December 2003 – 12 October 2005 | Togo signs in 2003 and ratifies without reservation in October 2005 the Maputo Protocol |
| Operational | Law N°. 2007-005 | 10 January 2007 | This law authorises VTP on prescription by a physician in cases of rape, incest, danger to the mother or high risk of serious illness for the unborn child. It has been inapplicable for 18 years. |
| Legislation | Public Health Code | 2009 | Article 107 prohibits the sale or distribution of methos or materials necessary for abortion. Article 108 authorizes pregnancy termination to save the life of a mother threatened by the continuation of pregnancy (certification by three physicians) or in cases of incest or rape (certified by three physicians) |
| Operational | National Policy for Gender Equity and Equality (PNEEG) | January 2011 | The Togolese government adopts a national policy for gender equality |
| Operational | National Strategy to Combat Gender-Based Violence | June 2012 | This strategy aims to reduce socio-cultural and economic inequalities within families and in society, to establish a legal and institutional environment conducive to non-violence |
| Legislation | Code of Medical Ethics | 2013 | Article 22 governs conscientious objection by healthcare professionals. Il specifies that a physician may only perform VTP under legal conditions, particularly when the continuation of pregnancy threatens the health or life of the mother or child |
| Legislation | New Penal Code | 2015 | Article 829 reaffirms the legal conditions for access to VTP. The new Code prohibits violence against women, including domestic violence and sexual assault |
| Legislation | Law N°. 2022-020 on the protection of learners against sexual violence in Togo | 2 December 2022 | This law strengthens the 1984 law on learner protection by covering all forms of sexual violence. It emphasizes support for victims and provides for the creation of a national observatory to monitor sexual violence in learning settings |
| Operational | National Protocol for the Care of GBV Victims | September 2023 | This protocol harmonizes procedures and guarantees victims access to integrated, quality services, without mentioning legal abortion in cases of sexual violence. |

Source: Field date, November 2024 – June 2025
